# DArT markers: diversity analyses and mapping in *Sorghum bicolor*

**DOI:** 10.1186/1471-2164-9-26

**Published:** 2008-01-22

**Authors:** Emma S Mace, Ling Xia, David R Jordan, Kirsten Halloran, Dipal K Parh, Eric Huttner, Peter Wenzl, Andrzej Kilian

**Affiliations:** 1 The Department of Primary Industries & Fisheries, Queensland (DPI&F), Hermitage Research Station, Warwick, QLD 4370, Australia; 2 Diversity Arrays Technology P/L, PO Box 7141, Yarralumla ACT 2600, Australia; 3 School of Land and Food Sciences, University of Queensland, Brisbane, QLD 4072, Australia; 4 Bureau of Sugar Experiment Station, DNRP, 50 Meiers Road, Indooroopilly, QLD 4068, Australia

## Abstract

**Background:**

The sequential nature of gel-based marker systems entails low throughput and high costs per assay. Commonly used marker systems such as SSR and SNP are also dependent on sequence information. These limitations result in high cost per data point and significantly limit the capacity of breeding programs to obtain sufficient return on investment to justify the routine use of marker-assisted breeding for many traits and particularly quantitative traits. Diversity Arrays Technology (DArT™) is a cost effective hybridisation-based marker technology that offers a high multiplexing level while being independent of sequence information. This technology offers sorghum breeding programs an alternative approach to whole-genome profiling. We report on the development, application, mapping and utility of DArT™ markers for sorghum germplasm.

**Results:**

A genotyping array was developed representing approximately 12,000 genomic clones using *Pst*I+*Ban*II complexity with a subset of clones obtained through the suppression subtractive hybridisation (SSH) method. The genotyping array was used to analyse a diverse set of sorghum genotypes and screening a Recombinant Inbred Lines (RIL) mapping population. Over 500 markers detected variation among 90 accessions used in a diversity analysis. Cluster analysis discriminated well between all 90 genotypes. To confirm that the sorghum DArT markers behave in a Mendelian manner, we constructed a genetic linkage map for a cross between R931945-2-2 and IS 8525 integrating DArT and other marker types. In total, 596 markers could be placed on the integrated linkage map, which spanned 1431.6 cM. The genetic linkage map had an average marker density of 1/2.39 cM, with an average DArT marker density of 1/3.9 cM.

**Conclusion:**

We have successfully developed DArT markers for *Sorghum bicolor *and have demonstrated that DArT provides high quality markers that can be used for diversity analyses and to construct medium-density genetic linkage maps. The high number of DArT markers generated in a single assay not only provides a precise estimate of genetic relationships among genotypes, but also their even distribution over the genome offers real advantages for a range of molecular breeding and genomics applications.

## Background

Sorghum is a major staple food and fodder crop grown worldwide, with an annual average production of 61 million tonnes over the past decade [1]. The crop is tolerant of many biotic and abiotic stresses and is often grown in more marginal cropping areas. In developing countries it tends to be a staple food and forage of the poor. In developed countries it is used primarily as an animal feed. Sorghum is often preferentially grown in both situations as it is better adapted to water limited environments than other cereal crops.

Investment in sorghum breeding and genomic resources has been less than for the other major cereals rice, wheat, maize and barley. Interest has focused on the crop due to its drought resistance and small genome size (~760 Mb) compared to close relatives maize (~2500 Mb) and sugarcane (2550 to 4200 Mb) [2-4]. In recent years the potential of sorghum as a biofuel crop has led to additional investment culminating in the sequencing of the sorghum genome [5].

Numerous studies have demonstrated that sorghum is very diverse crop, with cultivated sorghums exhibiting great phenotypic variability. The cultivated germplasm has been classified into five major races (bicolor, caudatum, durra, guinea and kafir) and 10 intermediate races based on panicle and spikelet morphology [6]. In order to exploit this diversity at the genotypic level, an efficient marker system is required. Many molecular marker technologies have been developed and applied to studying patterns of genetic diversity in sorghum germplasm collections and in breeding programs, including RFLPs [7-9], RAPDs [10,11], ISSRs [12], SSRs [13-19] and AFLPs [17,20]. However, the major limitation to the widespread use of current marker technologies in applied sorghum breeding programs and germplasm collections is the high cost per data point. Applications that require whole genome scans such as pedigree analysis [21], association mapping [22] and mapping as you go (MAYG) [23], or large scale genotyping of germplasm collections [24] are not cost effective using current technologies.

The current molecular marker technologies have characteristics which additionally affect the level of genome coverage, their discrimination ability, reproducibility and technical and time demand. A number of the limitations associated with the different marker technologies can be overcome by utilising specialised hardware such as high throughput capillary electrophoresis machines, which can impact on discrimination ability, reproducibility and speed. However, the majority of the limitations are related to the sequential nature and high assay costs of the marker technologies, in addition to reliance on DNA sequence information. Diversity arrays technology (DArT) can over come these limitations and has been developed as a hybridisation-based alternative to the majority of gel-based marker technologies currently in use. The DArT genotyping method was developed originally for rice [25] and has subsequently been applied to many other plant species, including barley [26], cassava [27], Arabidopsis [28], pigeonpea [29] and wheat [30]. DArT has been also applied to a number of animal species and microorganisms [31]. The DArT methodology offers a high multiplexing level, being able to simultaneously type several thousand loci per assay, while being independent of sequence information. DArT assays generate whole-genome fingerprints by scoring the presence versus absence of DNA fragments in genomic representations generated from genomic DNA samples through the process of complexity reduction.

This paper reports the results of a study designed to (1): develop a sorghum diversity array for DArT genotyping, (2): determine linkage map positions of polymorphic DArTs and (3): assess utility of DArT technology in diversity analyses on a set of diverse sorghum lines, including selected lines from the Department of Primary Industries and Fisheries (DPI&F) sorghum breeding program. In order to evaluate the discriminatory ability of DArTs, efforts have been made to include the same genotypes used in genetic diversity studies based on other molecular marker technologies.

## Results

### Evaluation of complexity reduction methods and array development

The initial tests of DArT performance in sorghum were done on eight sorghum genotypes (Additional File 1). Based on our positive experience with *Pst*I-based genomic representations [25] we initially evaluated several combinations of *Pst*I with different frequently cutting restriction enzymes (RE) as a complexity reduction approach for sorghum. DNA samples from the eight sorghum genotypes were digested with *Pst*I and several frequently cutting RE (*Pst*I+*Taq*I, *Pst*I+*Mse*I, *Pst*I+*Apo*I, *Pst*I+*Alu*I, *Pst*I+*Ban*II, *Pst*I+*Bst*NI and *Pst*I+*Afl*III), ligated to a *Pst*I adapter and then amplified with the *Pst*I-0 primer. Gel analysis of the PCR products showed that a uniform smear (without major bands) only appeared in the *Pst*I+*Ban*II combination. Other RE combinations gave a smear with one or more dominant bands. Such strong bands represent highly amplified restriction fragments and correspond to abundant repetitive sequences in the representation; a feature which is highly detrimental to DArT performance [32].

AFLP-like analysis was performed to estimate the fragment number in the *Pst*I+*Ban*II representation following the approach utilized by Xia et al. [27]. Four primers containing 3 selective bases at the 3'end were used for the amplification. For all four primers, a large number of fragments were visible on the gel, making a precise estimate of the total number of fragments impossible (data not presented). Interestingly, a similar approach resulted in an easily quantifiable number of bands in cassava which has a similar genome size to sorghum and even in barley, which has genome size almost seven times larger than sorghum. The results suggest that the *Pst*I+*Ban*II representation in sorghum has a larger number of unique fragments than the *Pst*I+*Bst*NI representation in barley which was reported to contain 1,546 markers placed on the integrated map of barley genome [26].

Based on the extended *Pst*I+*Ban*II sub-libraries and the additional libraries generated based on the genomic subtraction (SSH) method [33], the best DArT markers from the initial experiments were "cherry picked" and a "re-array library" with 768 polymorphic clones was created. In addition to these 768 polymorphism-enriched clones, a further 5367 clones from *Pst*I+*Ban*II Library C were used for all genotyping work reported here.

### Genetic relationships between sorghum lines revealed by DArT

The selected DArT clones were tested for their ability to resolve genetic relationships among a set of 90 lines. The reproducibility of the DArT genotyping array was successfully validated by independent assays from the same DNA. The genotypes selected represent a significant proportion of the genetic variation in sorghum with all 5 races represented and 5 intermediate races also represented. In addition, the germplasm set included elite lines from breeding programs some of which had high levels of co-ancestry.

DArTsoft analysis (see Materials and Methods) identified 508 markers polymorphic among 90 genotypes typed on the array. The PIC values of these 508 markers were very high with over 69% of the markers having a PIC value between 0.4 and 0.5 (Table 1). The average PIC was 0.41, higher than the previous DArT studies in barley (0.38 [26]) and comparable with cassava (0.42 [27]). The relationship between the quality of the DArT markers (measured as the % of total variance which existed between the two clusters: present and absent) and the performance of the DArT markers as determined through call rate and PIC was analysed (Table 2). The PIC values were largely independent of marker quality, with only a small reduction in PIC value observed in the lowest quality class (0.40 in the lowest quality class vs. 0.44 and 0.42 for the two higher quality classes). As expected, the average call rate decreased with average Q value. The markers with the highest Q values (above 90% of total variance between the clusters) had very high average call rates (98%), while the markers in the lower quality marker classes had lower average call rates and higher standard deviations.

**Table 1:** Polymorphism Information content (PIC) values for 508 DArT markers

PIC value	# DArTs	% DArTs
0.5-0.4	353	69.5
0.4-0.3	82	16.1
0.3-0.2	46	9.1
0.2-0.1	21	4.1
0.1-0	6	1.2

**Table 2:** The relationship between the quality and the performance of the DArT markers

	100 > Q > 90	90 > Q > 80	80 > Q > 70
Number of markers	37	236	235
Call Rate	98.03 ± 1.69	92.75 ± 3.51	88.01 ± 4.83
PIC	0.44 ± 0.08	0.42 ± 0.09	0.40 ± 0.11

**Figure 1:**
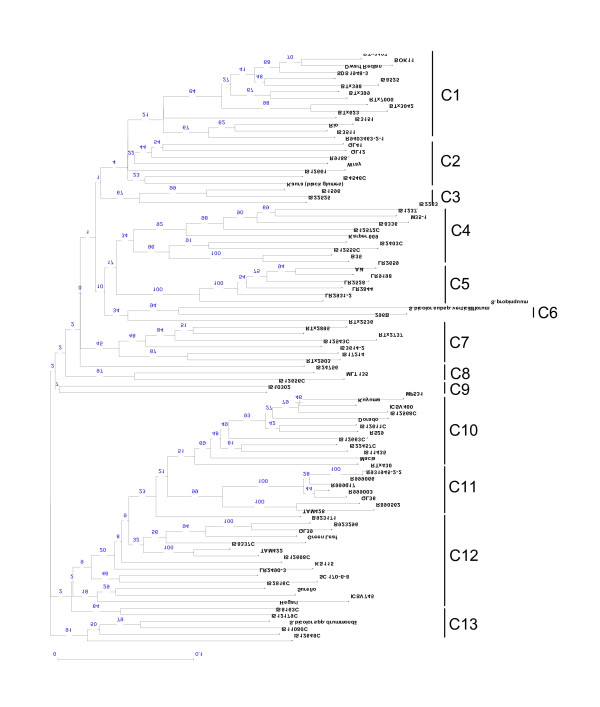
**Neighbor-joining anlaysis of diverse sorghum genotypes based on 508 DArT markers using the DICE similarity coefficient**. 13 clusters have been defined. The numbers on the branches indicate bootstrap values (expressed in percentages; based on 100 replications).

Cluster analysis based on the DICE dissimilarity index and the unweighted neighbour-joining method was performed on the 508 DArT markers for 90 genotypes (Figure 1). This cluster analysis discriminated well between all 90 genotypes and has a cophenetic correlation value of 0.9308, indicating an excellent fit of the similarity matrix data to the tree topology. Thirteen main clusters were identified which correspond well with race and origin of the genotyped lines. In particular a single predominant race or origin could be identified in 9 out of the 13 clusters. Cluster 1 contained 13 genotypes in total, of which 11 were kafir or kafir-caudatum. Cluster 4 contained 9 genotypes, of which 5 were of race durra. Additionally, two Ethiopian durra types (IS 12555C and B35) grouped together within this cluster with a boot strap value of 100%. Cluster 5 consisted of 6 Chinese genotypes of complex racial background. Cluster 6 contained the wild species, *S. propinquum *and the weedy subspecies, *S. bicolor *subsp. *verticilliflorum *(formerly *S. arundinaceum*). Cluster 9 consisted of 2 caudatum genotypes (IS 12656C and IS 10302). Cluster 10 consisted of 11 genotypes in total, of which 8 were caudatum or caudatum-derived. Cluster 11 consisted of a tight cluster of restorer (R) lines predominately from Australian breeding programs; R9990066, R999017 and R999003 are all progeny of the line R31945-2-2. Cluster 12 is a looser cluster consisting of 15 lines of which 7 were caudatum or caudatum-derived. Finally cluster 13 consisted of 4 genotypes of which 2 are bicolor or bicolor intermediate and *S. bicolor *subsp. *drummondii *which is very similar morphologically to the bicolor race.

### Mapping of DArT loci

To confirm that the sorghum DArT markers behave in a Mendelian manner, we constructed a genetic linkage map for a cross between R931945-2-2 and IS 8525. We selected 370 DArT markers with Q-values greater than 80% and merged with the segregation data for 286 markers, consisting of 55 SSRs, 229 AFLPs and 2 morphological markers derived from the original map [34].

**Figure 2:**
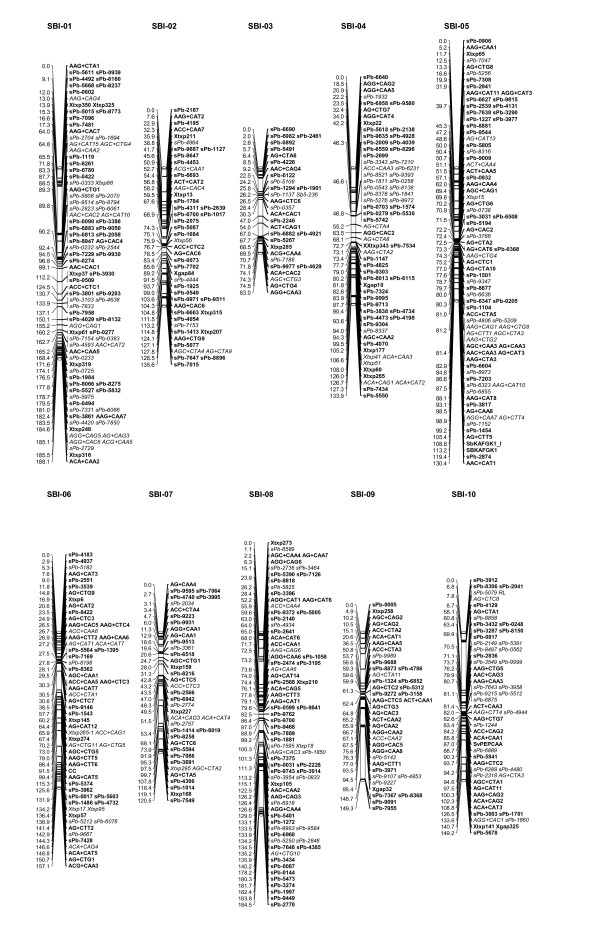
**Genetic linkage map for a cross between R31945-2-2 and IS 8525**. Genetic distances are expressed as cumulative map distances from position 0.0 (first locus of LG) in cM (Kosambi estimates). Locus names in bold indicate framework markers; locus names in italics indicate attached and delegate markers.

In total, 596 markers could be placed on the integrated linkage map, which spanned 1431.6 cM (Table 3). The genetic linkage map had an average marker density of 1/2.39 cM, with an average DArT marker density of 1/3.9 cM. The 358 DArT markers included on the map mapped to all 10 chromosomes and are distributed across the genome in a similar way as the 47 SSR and 188 AFLP markers (Figure 2), suggesting that the density of both groups of markers roughly followed the distribution of DNA polymorphism across the genome. The DArT markers accounted for approximately 50% of the framework markers used in the initial construction of each linkage group, with a high proportion of the DArT markers acting as delegates (Table 3; see M&M for explanations of framework, delegate and attached markers) indicating a higher level of redundancy compared to the other marker types. We therefore compared the level of redundancy, as determined through co-location, between the DArT markers generated from the 6 subtraction libraries (SSH) versus those developed from the extended *Pst*I+*Ban*II sub-libraries. Of the 358 DArTs included on the map, 172 were SSH-dervied and of these 50 (29%) were redundant, whereas the 186 non-SSH derived DArTs exhibited a reduced level of redundancy (26%). Overall redundancy in the map dropped from 33.7% to 24% when SSH-derived DArT markers were excluded. Interestingly, the difference in apparent redundancy levels between SSH-derived and "normal" DArT markers was smaller compared to what we observed for tomato and sugarcane (DArT P/L, unpublished) and in fern species *Asplenium *and moss species *Garovaglia *(DArT P/L and collaborators, unpublished data).

**Table 3:** Summary of the genetic linkage map based on a cross between R31945-2-2 and IS 8525. The genetic linkage map was constructed using DArTs, AFLPs and SSRs. The total length of each chromosome, the total number of markers, the total number of DArTs and the number of framework, delegate and attached markers per chromosome are detailed. For the last three columns, the number of DArTs in each class is given in parentheses

SBI	Length (cM)	Total # markers	# DArTs	# framework*	# delegate*	# attached*
1	188.1	94	65	31 (16)	25 (24)	38 (25)
2	135.6	48	31	31 (19)	9 (9)	9 (3)
3	83	31	19	21 (11)	4 (4)	6 (4)
4	133.9	70	49	28 (16)	18 (18)	24 (15)
5	130.4	81	43	38 (17)	18 (13)	25 (13)
6	157.1	61	23	36 (13)	9 (5)	16 (5)
7	120.5	40	24	26 (15)	5 (5)	9 (4)
8	184.5	75	51	41 (26)	14 (11)	20 (14)
9	149.3	40	19	24 (7)	9 (7)	7 (5)
10	149.2	56	34	26 (9)	6 (5)	25 (20)

Totals	1431.6	596	358	302 (149)	117 (101)	179 (108)

* 'Framework' markers are defined as those that could be ordered with a jack-knife value of 90% or greater using the MultiPoint software; Delegate markers are defined as those that map to the same location as the representative framework marker for a specific locus; Attached markers are those that initially were excluded from the framework map as they caused unstable neighborhoods but were included in the final, complete map by assigning them to the best intervals on the framework map.

There was no statistically significant difference between DArT and non-DArT markers in the distribution of parental alleles across the genome. Twelve DArTs were removed during the course of map construction either because of lack of linkage to other markers, or as they formed small linkage groups containing only DArTs which did not link to the known chromosomes.

## Discussion

### Sorghum DArT markers

This is the first report of the use of DArT technology in sorghum and our results demonstrate that the sorghum DArT markers are high quality, as assessed by their call rate, scoring reproducibility and PIC values. The DArT marker quality parameters measured for the sorghum array are comparable to those obtained for pigeonpea [29], barley [26] and cassava [27] and wheat [30].

### Utility of DArTs for diversity analysis

Among the criteria for genetic markers that are to be used for fingerprinting and marker-assisted selection is a high level of polymorphism [15]. Clearly, sorghum DArTs meet this criterion, with over 69% of the 508 DArTs revealing polymorphism between the set of 90 sorghum lines having a PIC value between 0.5-0.4; 0.5 being the highest PIC value expected for a bi-allelic marker system. Additionally, this study meets all the criteria proposed for evaluating the use of molecular markers for large scale germplasm diversity analyses [35]; a large number of markers, thorough representation of these markers on a genetic linkage map of the species and the selection of monolocus probes. The 508 DArT markers permitted the unique identification of all 90 genotypes including closely related backcross derived lines. The cluster analysis revealed that the 90 genotypes examined showed a clear demarcation of the germplasm according to their racial classification, consistent with previous studies using RFLPs [9] and SSRs and AFLPs [17]. The bicolor race was found to be highly variable in this study, shown by its presence in multiple clusters, as also noted by previous studies [8,9,14]. In fact, it has been noted previously that race bicolor resembles spontaneous weedy sorghums and is thought to be the race most closely related to wild sorghums [18] and also the most primitive grain sorghum [10]. Indeed, the bicolor race accession IS 12179C included in this study, groups together with the weedy sorghum, *S. bicolor *subsp. *drummondii*, in cluster 13 (Figure 1).

The race caudatum is one of the most important agronomically, providing genes for high yield and excellent seed quality. It has become one of the most important sources of germplasm in modern breeding programs throughout the world and in this study, the caudatum race genotypes were clearly demarcated in clusters 9, 10 and 12. In contrast, a previous study [18] found no significant differences in diversity between the races, except the kafir race which was less diverse. Low genetic diversity was also observed in the kafir race in this study, with all the kafir race accessions constituting a specific cluster (cluster 1). These results are in agreement with the recent origin and restricted geographic distribution of this race, together with recent studies using SSRs [14] and RFLPs [9].

In addition to the observed clustering on racial groups, differences between the genotypes based on their status as B (maintainer female) and R (male parental restorer) lines was also noted. In this study, there was less variation among the B-lines than the R-lines, with the B-lines clustering tightly together in only 2 of the 13 clusters, whereas the R-lines grouped more loosely in 5 clusters. It has also been noted [17] that the lower levels of diversity among elite B-lines vs. R-lines is expected since B-lines are required to produce high quality male-sterile A-lines. The development of new A/B-lines is more difficult compared to R-line development and hence B-line development is more restrictive and slower to incorporate new germplasm. Where pedigree data was available, groupings associated with these pedigree relationships were observed.

Comparisons of the discrimination ability for DArT markers and their ability to reflect pedigree backgrounds in contrast to other marker types is made much simpler when over-lapping sets of germplasm are used in a number of studies. Seventeen genotypes included in this study were examined by Menz et al. [17], 54 genotypes were in common with Ritter et al. [20] and 11 with Tao et al. [7]. Very similar groupings of genotypes were observed, e.g. the over-lapping genotypes in cluster 1 also group together in previous studies [17,20], e.g. BTx3197 and BOK11 grouping tightly together in Menz et al. [17], in addition to RTx7000 and Btx3042. Similarly, ICSV400, MP531 and Macia group together based on AFLPs [17,20] and DArTs (cluster 10), and QL41 and R9188 group together based on RFLPs [7] and DArTs (cluster 2).

The separation of "wild" sorghums from cultivated germplasm seems to be less pronounced compared to the results based on SSR data [18]. This is not surprising, as the array used for genotyping all materials in this study was developed using mostly DNA from cultivated materials. Even though three accessions from wild relatives were included in library construction, their "private" alleles were highly "diluted" by common alleles and those which were "private" to the cultivated material. The design of the array did not substantially change the topology of trees generated from DArT data, but would reduce the distance between the wild and cultivated samples. We expect that the distance reduction would be up to two-fold, as we were effectively finding unique "0" scores for the wild materials, but less effectively unique "1" scores. Such ascertainment bias is not limited to DArT, but is a feature of many marker systems. In fact the ascertainment bias will be significantly larger for SNP technologies, as most SNP markers are developed from a small number of samples/chromosomes in a limited number of populations, even in well resourced human studies [36,37]. The effect of such "marker source sampling bias" was recognised in many human studies, e.g. on population migration rate [38], population mutation and recombination rate estimates [39] and on Linkage Disequilibrium (LD) estimates [40]. Marker panels in DArT are developed using large and representative sampling from target populations [32] and can be easily expanded by cloning libraries from the germplasm pools for which precise relatedness estimates would be required.

### Distribution of DArT loci in the sorghum genome

The integrated genetic linkage map comprising DArTs, AFLPs and SSRs clearly demonstrates that the new sorghum DArT markers behave in a Mendelian manner. In total, 358 DArTs were mapped to 257 unique loci. The higher level of redundancy of the DArT markers is reflected by the higher number of AFLP and SSR markers having unique segregation patterns. However, the markers on the DArT array used in this study were not filtered for redundancy, whereas the SSR/AFLP data set had previously undergone curation [34], to remove markers with redundant segregation patterns. Also, the total number of DArT markers was higher than in other marker classes, therefore the apparent redundancy would need to be also corrected for sample size. After applying such sample-size correction to STMP markers in the linkage map of a cross between two wheat cultivars Cranbrook and Halberd, a lower level of redundancy was found for the DArT markers [30].

The total length of the integrated genetic linkage map was 1431.6 cM, with an average DArT marker density of 1 per every 3.9 cM. The total map length is comparable to other recently reported sorghum genetic maps, being slightly shorter than the 1713 cM high-density genetic linkage map based on 2926 AFLP, RFLP and SSR markers [41], and slightly longer than the 1059.2 cM genetic linkage map based on 2050 RFLP probes [42]. Although the DArT markers are distributed across the genome in a similar way to the non-DArT markers, there are genomic regions containing significant excesses or paucity of markers, e.g. the centromeric region of SBI-05 has a higher than average marker density of 1/0.64 cM and the distal ends of SBI-01 and SBI-10 have marker-poor regions with gaps spanning over 30 cM. These areas of low marker density may correspond to regions of similar ancestry or identity by descent (IBD) in the germplasm included in the initial diversity representation. In addition, lines with photoperiod sensitivity and tall stature were under represented in the diversity set used to develop the DArT markers. These regions of low marker density may be therefore associated with genomic regions that were identical by descent or that had very limited genetic variability in the initial diversity representation. The marker-dense regions appear to correspond to the centromeric regions, a feature that has been observed previously [42]. This is also supported by the recent observation that the pericentromeric heterochromatic regions of sorghum chromosomes show much lower rates of recombination (~8.7 Mbp/cM) compared to euchromatic regions (~0.25 Mbp/cM), with the average rate of recombination across the heterochromatic portion of the sorghum genome being ~34-fold lower than recombination in the euchromatic region [43]. It should however be noted that clustering around the centromeres is observed for both DArT and non-DArT markers, due to the centromeric suppression of recombination. Interestingly, DArT markers were significantly less clustered at most centromeric regions of barley chromosomes compared to non-DArT markers on the integrated map containing approximately 3,000 markers [26]. We will be in position to rigorously test if this difference in marker position holds true in sorghum only after completion of building the consensus map integrating approximately 1,000 DArT markers with similar number of other types of markers [Mace et al in preparation].

## Conclusion

We have successfully developed DArT markers for *Sorghum bicolor *and have demonstrated that DArT provides high quality markers that can be used for diversity analyses and to construct medium-density genetic linkage maps. The high number of DArT markers generated in a single assay not only provides a precise estimate of genetic relationships among genotypes, but also their even distribution over the genome offers real advantages for a range of molecular breeding and genomics applications. Additionally, the availability of the sorghum whole genome sequence by the end of 2007 offers very exciting opportunities for assessing the colinearity of the DArT markers on the genetic linkage maps with the markers on the sequence map. As DArT assays are performed on highly parallel and automated platforms the cost of datapoint (a few cents per marker assay) is reduced by at least an order of magnitude compared to current, gel-based technologies.

## Methods

### Source of DNA

The sorghum accessions used to prepare DArT libraries represent the genetic diversity present in the cultivated species (*S. bicolor *subsp. *bicolor*) with all 5 races and 5 intermediate races represented, two weedy subspecies (*S. bicolor *subsp. *drummondii *and subsp. *verticilliflorum*) and a wild species, *S. propinqum *(Additional File 1). In addition, the germplasm set included elite lines from breeding programs some of which had high levels of co-ancestry. DNA was extracted using a modified CTAB-based extraction protocol [44,45].

### Development of DArT for sorghum

Several DArT arrays were built in the course of this study. For each of these arrays, a genomic representation was generated from a mixture of sorghum lines using the *Pst*I-based complexity reduction method previously described [26]. Libraries were prepared as described previously [25]. Two extended *Pst*I+*Ban*II sub-libraries were subsequently generated using DNA from 31 and 94 genotypes, respectively (Additional File 2). In addition, six libraries were generated by applying the SSH method to genomic representations [33]. Drivers and testers used in the subtraction libraries construction were as shown in Table 4. DNA of drivers and testers was digested by *Pst*I/*Bst*NI and ligated to the *Pst*I adaptor. The digestion/ligation products were amplified using the *Pst*I-0 primer. The resulting PCR products were digested with a RE mixture containing *Dpn*II, *Hpy*CH4IV, *Mse*I and *Nla*III. Subtraction was done in a 30:1 ratio of driver to tester and carried out in one and two rounds of subtractive hybridization. After final amplification, the subtraction products were cloned as detailed previously [27]. The sorghum libraries utilized for the initial marker discovery are summarized in the supplementary material (Additional File 3). Clones from all libraries with the exception of *Pst*I+*Ban*II Library C were used to create arrays in order to genotype several hundred sorghum accessions (data not presented). The best markers from the initial experiments were "cherry picked" to assemble the "re-array library" with 768 polymorphic clones. Clones from this library together with 5367 clones from *Pst*I+*Ban*II Library C were used for all genotyping work reported here. The re-array library was created using the *Pst*I+*Ban*II and the subtraction (SSH) libraries. Details of the re-array library and other libraries used for sorghum genotyping are included in the supplementary material (Additional Files 2 &3).

**Table 4:** Drivers and testers used in the subtraction libraries

	Library 1	Library 2	Library 3	Library 4	Library 5	Library 6
Subtraction method	One round	One round	One round	One round	One round	Two rounds
Library	Subtraction-1	Subtraction-2	Subtraction-3	Subtraction-4	Subtraction-5	Subtraction-6
Driver	ISCV745	ISCV745	90562	IS8525	31945-2-2	Mixture of drivers
tester	Tester mixture*	90562	ISCV745	31945-2-2	IS8525	Tester mixture*

* The tester mixture was comprised of 90 sorghum genotypes without the four parental genotypes (ICSV745, B90562, R31945-2-2 and IS 8525).

### DArT genotyping

Genotyping was performed essentially as described in references 26 and 30. Briefly, each genomic DNA sample is subjected to the PstI+BanII complexity reduction method. The resulting genomic representation is labelled with fluorescent nucleotides and hybridised on a microarray printed with the DArT clones. A typical experiment is performed on about 94 samples of genomic DNA. Following hybridisation and washing, the microarrays for an experiment are scanned, the images are analysed and the score of each marker is calculated for each sample by dedicated software DArTsoft: markers are scored 1 for presence, 0 for absence and X for inability to score. The quality parameter Q for each marker is calculated by dividing the variance of the hybridisation level for the marker between the 2 clusters (present and absent) by the total variance of hybridisation level of the marker, in the experiment.

### Diversity analysis

A group of 90 sorghum lines (subset detailed in Additional File 1) were genotyped on the re-array library as described previously [30]. The sorghum lines were chosen to provide a reasonable representation of sorghum genetic diversity as well as including elite inbred lines from the DPI&F and other sorghum breeding programs. Some of the elite lines were quite closely related and were included to demonstrate the discrimination possible with DArT.

The marker scores were subjected to cluster and principal coordinate analysis using the DARwin [46] to visualize the genetic relationships among the lines. Additionally, the polymorphism information content (PIC) of each DArT marker was determined as follows; PIC = 1-ΣP_i_^2^, where P_i _is the frequency of the *i*th allele in the examined genotypes [47].

### Genetic mapping

The lines R931945-2-2, a commercially accepted restorer line in Australia and IS 8525, an Ethiopian line (kafir race), in addition to 92 lines of a F_5 _recombinant inbred line (RIL) population derived from a cross between the two lines were typed using the genotyping array. Clones with Q > 80% and a call rate of at least 80% were selected for mapping. DArTs markers were merged with an existing mapping data set consisting of 286 markers including 55 SSRs and 229 AFLPs [34]. A genetic linkage map was constructed using MultiPoint software [48]. The RIL_Selfing population setting was selected and a maximum threshold rf_s _value of 0.40 was used to initially group the markers into ten linkage groups. Multipoint linkage analysis of loci within each LG was then performed and marker order was further verified through re-sampling for quality control via jack-knifing [49]. Markers that could be ordered with a jack-knife value of 90% or greater were included as 'framework' markers, with any remaining markers causing unstable neighborhoods being initially excluded from the map, including redundant markers mapping to the same location. Following a repeated multipoint linkage analysis with the reduced set of markers for each LG to achieve a stabilised neighbourhood, the previously excluded markers were attached by assigning them to the best intervals on the framework map and labelled as attached markers. The redundant markers were also included on the final, complete map but labelled as delegates. Finally, the linkage groups were assigned to sorghum chromosomes, SBI-01 to SBI-10 according to recent nomenclature [50]. The Kosambi [51] mapping function was used to calculate the centimorgan (cM) values. The graphical representation of the map was drawn using MapChart software [52].

## Authors' contributions

ESM carried out the diversity and mapping analyses and drafted the manuscript. LX co-developed the DArT technology for sorghum. DRJ conceived of the study, and participated in its design and coordination, germplasm development and selection and helped to draft the manuscript. KH was involved in the optimisation of the methodology and participated in the mapping analysis. DKP participated in the mapping analysis. EH participated in the development of the DArT technology and editing of the manuscript. PW contributed to ongoing improvements of the DArT technology and the quality assessment of sorghum clones. AK supervised development of the DArT technology, participated in the study's design and coordination and helped to draft the manuscript.

## Supplementary Material

Additional File 1 List of sorghum genotypes used for the development of the sorghum DArT array and the diversity analyses. The table includes details of genotype IDs and aliases, race and origin and inclusion status in both the methodology developmental stages and diversity analyses. Click here for file

Additional File 2 Summary of sorghum libraries. The table includes details of the number of genotypes used in the development of each sorghum library and the number of clones identified. Click here for file

Additional File 3 Libraries used for generation of the genotyping array. The table details the barcode for each sorghum library. Click here for file
