# Supplementary material for: DArT markers: diversity analyses and mapping in Sorghum bicolor
Source: BMC Genomics. 2008 Jan 22;9:26. doi: 10.1186/1471-2164-9-26 (PMC2270266; doi:10.1186/1471-2164-9-26)
Supplement: Additional File 1 — List of sorghum genotypes used for the development of the sorghum DArT array and the diversity analyses. The table includes details of genotype IDs and aliases, race and origin and inclusion status in both the methodology developmental stages and diversity analyses. [file 1471-2164-9-26-S1.doc]

**Additional File 1. List of sorghum genotypes used for the development of the DArT array and diversity analyses**

|  |  |  |  |  |  | PstI+BanII library | | | | Subtraction |
| --- | --- | --- | --- | --- | --- | --- | --- | --- | --- | --- |
| **No** | **Code** | **Aliases** | **Race** | **Origin** | **Diversity set** | initial test | library A | library B | library C | library 1-6 |
| 1 | QL39 |  | complex | Australian breeding program | ✓ |  |  | ✓ | ✓ | ✓ |
| 2 | QL41 |  | complex | Australian breeding program | ✓ |  |  | ✓ | ✓ | ✓ |
| 3 | R890562 | R90562 | complex | Australian breeding program | ✓ |  |  | ✓ | ✓ | ✓ |
| 4 | ICSV 745 |  | complex | ICRISAT breeding program | ✓ |  |  | ✓ | ✓ | ✓ |
| 5 | R931945-2-2 | R31945-2-2 | complex | Australian breeding program | ✓ |  |  | ✓ | ✓ | ✓ |
| 6 | *S. bicolor* subsp. *verticilliflorum* | *S. arundinaceum* | Wild relative | endemic Australia | ✓ |  |  | ✓ | ✓ | ✓ |
| 7 | IS 8525 | PI 563092 | kafir | Ethiopia | ✓ |  |  | ✓ | ✓ | ✓ |
| 8 | B923296 | B23296 | complex | Australian breeding program | ✓ |  |  | ✓ | ✓ | ✓ |
| 9 | SC 170-6-8 | Partially converted IS 12661 | caudatum | Ethiopia | ✓ | ✓ |  | ✓ | ✓ | ✓ |
| 10 | B35 | BTx642 | durra | Ethiopia | ✓ | ✓ |  | ✓ | ✓ | ✓ |
| 11 | QL12 |  | complex | Australian breeding program | ✓ | ✓ |  | ✓ | ✓ | ✓ |
| 12 | R9403463-2-1 | R403463-2-1 | complex | Australian breeding program | ✓ |  |  | ✓ | ✓ | ✓ |
| 13 | R9188 |  | caudatum-bicolor | unknown | ✓ |  |  | ✓ | ✓ | ✓ |
| 14 | BTx623 |  | kafir-caudatum | USA breeding program | ✓ |  | ✓ | ✓ | ✓ | ✓ |
| 15 | QL36 |  | complex | Australian breeding program | ✓ |  |  | ✓ | ✓ | ✓ |
| 16 | B923171 | B23171 | complex | Australian breeding program | ✓ |  |  | ✓ | ✓ | ✓ |
| 17 | BKS4 |  | kafir | USA breeding program |  | ✓ |  | ✓ | ✓ | ✓ |
| 18 | BOK11 |  | kafir | USA breeding program | ✓ |  |  | ✓ | ✓ | ✓ |
| 19 | 296B |  | complex | China | ✓ |  |  | ✓ | ✓ | ✓ |
| 20 | BTx399 | Wheatland | kafir-caudatum | USA breeding program | ✓ |  |  | ✓ | ✓ | ✓ |
| 21 | BTx3197 | Combine Kafir 60 | kafir | USA breeding program | ✓ |  |  | ✓ | ✓ | ✓ |
| 22 | BTx3042 |  | kafir-caudatum | USA breeding program | ✓ |  |  | ✓ | ✓ | ✓ |
| 23 | BTx398 | Martin | kafir | USA breeding program | ✓ |  |  | ✓ | ✓ | ✓ |
| 24 | Dwarf Redlan |  | kafir-caudatum | USA breeding program | ✓ |  |  | ✓ | ✓ | ✓ |
| 25 | RTx2895 |  | complex | USA breeding program | ✓ |  | ✓ | ✓ | ✓ | ✓ |
| 26 | RTx2536 |  | durra-caudatum | USA breeding program | ✓ |  |  | ✓ | ✓ | ✓ |
| 27 | RTx7000 |  | kafir-caudatum | USA breeding program | ✓ |  |  | ✓ | ✓ | ✓ |
| 28 | TAM422 |  | durra-caudatum | USA breeding program | ✓ | ✓ |  | ✓ | ✓ | ✓ |
| 29 | RTx2737 |  | complex | USA breeding program | ✓ |  |  | ✓ | ✓ | ✓ |
| 30 | RTx2903 |  | complex | USA breeding program | ✓ |  | ✓ | ✓ | ✓ | ✓ |
| 31 | Ai4 |  | complex | China breeding program | ✓ | ✓ | ✓ | ✓ | ✓ | ✓ |
| 32 | LR9198 |  | complex | China breeding program | ✓ |  | ✓ | ✓ | ✓ | ✓ |
| 33 | LR2931-2 |  | complex | China breeding program | ✓ |  |  | ✓ | ✓ | ✓ |
| 34 | LR2528 |  | complex | China breeding program | ✓ |  |  | ✓ | ✓ | ✓ |
| 35 | LR2659 |  | complex | China breeding program | ✓ |  | ✓ | ✓ | ✓ | ✓ |
| 36 | LR2490-3 |  | complex | China breeding program | ✓ |  | ✓ | ✓ | ✓ | ✓ |
| 37 | LR2844 |  | complex | China breeding program | ✓ |  |  | ✓ | ✓ | ✓ |
| 38 | ICSV 400 |  | caudatum derivative | ICRISAT breeding program | ✓ |  | ✓ | ✓ | ✓ | ✓ |
| 39 | Kuyuma | WSV 387, SDS 3136-2 | complex | Collaborative ICRISAT/SADC-Zambia breeding program | ✓ |  | ✓ | ✓ | ✓ | ✓ |
| 40 | MP531 |  | caudatum derivative | USA breeding program | ✓ |  |  | ✓ | ✓ | ✓ |
| 41 | Sureño | M62650 | complex | ICRISAT breeding program | ✓ |  | ✓ | ✓ | ✓ | ✓ |
| 42 | SDS 1948-3 |  | complex | Collaborative ICRISAT/SADC breeding program | ✓ |  | ✓ | ✓ | ✓ | ✓ |
| 43 | Macia | M91057, SDS 3220 | caudatum derivative | ICRISAT/SADC breeding program | ✓ |  | ✓ | ✓ | ✓ | ✓ |
| 44 | RS29 |  | complex | Indian breeding program | ✓ |  | ✓ | ✓ | ✓ | ✓ |
| 45 | M35-1 | IS 1054 | durra | India | ✓ |  | ✓ | ✓ | ✓ | ✓ |
| 46 | Green Leaf | Green Leaf Sudan Grass, | bicolor | Indian Breeding program | ✓ |  | ✓ | ✓ | ✓ | ✓ |
| 47 | *S. bicolor* spp. *drummondii* | PI 330272, AusTRCF 300263 | Wild relative | Ethiopia | ✓ |  | ✓ | ✓ | ✓ | ✓ |
| 48 | *S. propinquum* |  | Wild relative | S.E. Asia | ✓ | ✓ | ✓ | ✓ | ✓ | ✓ |
| 49 | Wray |  | kafir derivative | USA breeding program | ✓ |  | ✓ | ✓ | ✓ | ✓ |
| 50 | Rio |  | kafir | USA breeding program | ✓ |  | ✓ | ✓ | ✓ | ✓ |
| 51 | IS 12611C | SC 111, PI 534146 | caudatum | Ethiopia | ✓ |  |  | ✓ | ✓ | ✓ |
| 52 | IS 22457C | SC 1552, PI 569903 | caudatum | Sudan | ✓ |  | ✓ | ✓ | ✓ | ✓ |
| 53 | IS 22525 | PI 569969 | caudatum | Sudan | ✓ |  | ✓ | ✓ | ✓ | ✓ |
| 54 | KS 115 |  | durra | unknown | ✓ |  |  | ✓ | ✓ | ✓ |
| 55 | MLT 135 |  | complex | USA breeding program | ✓ |  |  | ✓ | ✓ | ✓ |
| 56 | IS 12568C | SC 56-14E, PI 152702 | caudatum | Sudan | ✓ |  |  | ✓ | ✓ | ✓ |
| 57 | IS 12608C | SC 108C, PI 257595 | caudatum | Ethiopia | ✓ |  |  | ✓ | ✓ | ✓ |
| 58 | IS 2403C | SC 103-14E, PI 239441 | guinea-caudatum | South Africa | ✓ |  |  | ✓ | ✓ | ✓ |
| 59 | IS 12543C | SC 23C, PI 148084 | durra | Ethiopia | ✓ |  |  | ✓ | ✓ | ✓ |
| 60 | Karper 669 |  | complex | USA breeding program | ✓ |  |  | ✓ | ✓ | ✓ |
| 61 | IS 12648C | SC 157-6, PI 276818 | kafir-caudatum | Ethiopia | ✓ |  |  | ✓ | ✓ | ✓ |
| 62 | IS 12572C | SC 62C, PI 152730 | complex | Kenya | ✓ |  |  | ✓ | ✓ | ✓ |
| 63 | IS 4546C | SC 871-6, PI 644350 | durra | India | ✓ |  | ✓ | ✓ | ✓ | ✓ |
| 64 | IS 12601C | SC 92-4, PI 197242 | durra | Ethiopia |  |  |  | ✓ | ✓ | ✓ |
| 65 | IS 17214 | SC 1075, NSL 365700 | unknown | Nigeria | ✓ |  |  | ✓ | ✓ | ✓ |
| 66 | IS 3614-2 | NSL 50587 | guinea | Nigeria | ✓ |  |  | ✓ | ✓ | ✓ |
| 67 | IS 12663C | SC 172, PI 276839 | durra | Ethiopia | ✓ |  |  | ✓ | ✓ | ✓ |
| 68 | IS 8163C | SC 636-6, NSL 51495 | kafir-caudatum | India | ✓ |  |  | ✓ | ✓ | ✓ |
| 69 | IS 11080C | SC 999, PI 329323 | durra-bicolor | Ethiopia | ✓ |  |  | ✓ | ✓ | ✓ |
| 70 | IS 10302 |  | caudatum | Thailand | ✓ |  |  | ✓ | ✓ | ✓ |
| 71 | IS 11435 |  | caudatum | Ethiopia | ✓ |  |  | ✓ | ✓ | ✓ |
| 72 | IS 12179C |  | bicolor | Ethiopia | ✓ |  | ✓ | ✓ | ✓ | ✓ |
| 73 | IS 1237 | NSL 365680 | bicolor | India | ✓ |  | ✓ | ✓ | ✓ | ✓ |
| 74 | IS 1596 | PI 291238 | guinea | Jamaica | ✓ |  |  | ✓ | ✓ | ✓ |
| 75 | IS 2263 |  | durra | Sudan | ✓ |  |  | ✓ | ✓ | ✓ |
| 76 | IS 3151 |  | caudatum | South Africa | ✓ |  |  | ✓ | ✓ | ✓ |
| 77 | IS 3511 |  | kafir-caudatum | Sudan | ✓ |  |  | ✓ | ✓ | ✓ |
| 78 | IS 8336 |  | durra | Pakistan | ✓ |  |  | ✓ | ✓ | ✓ |
| 79 | Hegari |  | caudatum | unknown | ✓ | ✓ |  | ✓ | ✓ | ✓ |
| 80 | IS 12661 |  | caudatum | Ethiopia | ✓ |  |  | ✓ | ✓ | ✓ |
| 81 | TAM2566 | SC 175-9 | caudatum derivative | USA breeding program |  |  | ✓ | ✓ | ✓ | ✓ |
| 82 | TAM428 | SC 110-9 | caudatum derivative | USA breeding program | ✓ |  |  | ✓ | ✓ | ✓ |
| 83 | Dorado | Grif 642, ISIAP Dorado | caudatum | Collaborative ICRISAT/CENTA breeding program in El Salvador | ✓ |  | ✓ | ✓ | ✓ | ✓ |
| 84 | IS 24756 | Grif 9808 | unknown | Nigeria | ✓ |  | ✓ | ✓ | ✓ | ✓ |
| 85 | Kaura (black glumes) | Kaura (black glume) Nth Nig | caudatum derivative | Nigeria | ✓ |  |  | ✓ | ✓ | ✓ |
| 86 | IS 2816C | Zera-zera white, SC 120C | caudatum | Zimbabwe | ✓ |  |  | ✓ | ✓ | ✓ |
| 87 | IS 12656C | SC 165C, PI 533923 | caudatum | Ethiopia | ✓ |  |  | ✓ | ✓ | ✓ |
| 88 | IS 12555C | SC 35C | durra | Ethiopia | ✓ |  |  | ✓ | ✓ | ✓ |
| 89 | IS 8337C | SC 574C, NSL 54491 | caudatum | Pakistan | ✓ |  | ✓ | ✓ | ✓ | ✓ |
| 90 | R999003 |  | complex | Australian breeding program | ✓ |  |  | ✓ | ✓ | ✓ |
| 91 | R999017 |  | complex | Australian breeding program | ✓ |  |  | ✓ | ✓ | ✓ |
| 92 | R999066 |  | complex | Australian breeding program | ✓ |  |  | ✓ | ✓ | ✓ |
| 93 | RTx430 |  | complex | USA breeding program | ✓ |  | ✓ | ✓ | ✓ | ✓ |
