# Supplementary material for: DArT markers: diversity analyses and mapping in Sorghum bicolor
Source: BMC Genomics. 2008 Jan 22;9:26. doi: 10.1186/1471-2164-9-26 (PMC2270266; doi:10.1186/1471-2164-9-26)
Supplement: Additional File 2 — Summary of sorghum libraries. The table includes details of the number of genotypes used in the development of each sorghum library and the number of clones identified. [file 1471-2164-9-26-S2.doc]

**Additional File 2**. Summary of sorghum libraries

| library | Genotype number | clones |
| --- | --- | --- |
| *Pst*I+*Ban*II test library | 8 | 768 |
| *Pst*I+*Ban*II library A | 31 | 1536 |
| *Pst*I+*Ban*II library B | 94 | 5376 |
| *Pst*I+*Ban*II library C* | 94 | 5376 |
| Subtraction-1 library | 91 | 768 |
| Subtraction-2 library | 2 | 768 |
| Subtraction-3 library | 2 | 768 |
| Subtraction-4 library | 2 | 768 |
| Subtraction-5 library | 2 | 768 |
| Subtraction-6 library | 94 | 768 |
| Re-array library | 94 | 768 |

*The genotypes in library C are the same as in library B, but new DNA extracts were used.
