# Supplementary material for: DArT markers: diversity analyses and mapping in Sorghum bicolor
Source: BMC Genomics. 2008 Jan 22;9:26. doi: 10.1186/1471-2164-9-26 (PMC2270266; doi:10.1186/1471-2164-9-26)
Supplement: Additional File 3 — Libraries used for generation of the genotyping array. The table details the barcode for each sorghum library. [file 1471-2164-9-26-S3.doc]

**Additional File 3**. The libraries used for sorghum service

| Library | Barcode spotting plate |
| --- | --- |
| Re-array library | L/ling/8-8-05/001 |
| Re-array library | L/ling/1-8-05/001 |
| *Pst*I+*Ban*II library C | L/ling/28-7-05/001 |
| *Pst*I+*Ban*II library C | L/ling/28-7-05/002 |
| *Pst*I+*Ban*II library C | L/ling/28-7-05/003 |
| *Pst*I+*Ban*II library C | L/ling/28-7-05/004 |
| *Pst*I+*Ban*II library C | L/ling/28-7-05/005 |
| *Pst*I+*Ban*II library C | L/ling/28-7-05/006 |
| *Pst*I+*Ban*II library C | L/ling/28-7-05/007 |
| *Pst*I+*Ban*II library C | L/ling/28-7-05/008 |
| *Pst*I+*Ban*II library C | L/ling/28-7-05/009 |
| *Pst*I+*Ban*II library C | L/ling/28-7-05/010 |
| *Pst*I+*Ban*II library C | L/ling/28-7-05/011 |
| *Pst*I+*Ban*II library C | L/ling/28-7-05/013 |
| *Pst*I+*Ban*II library C | L/ling/28-7-05/014 |
